# Hidden regulators: the emerging roles of lncRNAs in brain development and disease

**DOI:** 10.3389/fnins.2024.1392688

**Published:** 2024-05-22

**Authors:** Farah Alammari, Ensaf M. Al-Hujaily, Alaa Alshareeda, Nada Albarakati, Batla S. Al-Sowayan

**Affiliations:** ^1^Department of Blood and Cancer Research, King Abdullah International Medical Research Center, Riyadh, Saudi Arabia; ^2^Clinical Laboratory Sciences Department, College of Applied Medical Sciences, King Saud Bin Abdulaziz University for Health Sciences, Riyadh, Saudi Arabia; ^3^King Saud Bin Abdulaziz University for Health Sciences, Riyadh, Saudi Arabia; ^4^Saudi Biobank Department, King Abdullah International Medical Research Center, Riyadh, Saudi Arabia; ^5^Department of Blood and Cancer Research, King Abdullah International Medical Research Center, Jeddah, Saudi Arabia; ^6^King Saud Bin Abdulaziz University for Health Sciences, Ministry of the National Guard-Health Affairs, Jeddah, Saudi Arabia

**Keywords:** long non-coding RNAs, neurogenesis, gene regulation, neuronal development, neurological disorders

## Abstract

Long non-coding RNAs (lncRNAs) have emerged as critical players in brain development and disease. These non-coding transcripts, which once considered as “transcriptional junk,” are now known for their regulatory roles in gene expression. In brain development, lncRNAs participate in many processes, including neurogenesis, neuronal differentiation, and synaptogenesis. They employ their effect through a wide variety of transcriptional and post-transcriptional regulatory mechanisms through interactions with chromatin modifiers, transcription factors, and other regulatory molecules. Dysregulation of lncRNAs has been associated with certain brain diseases, including Alzheimer’s disease, Parkinson’s disease, cancer, and neurodevelopmental disorders. Altered expression and function of specific lncRNAs have been implicated with disrupted neuronal connectivity, impaired synaptic plasticity, and aberrant gene expression pattern, highlighting the functional importance of this subclass of brain-enriched RNAs. Moreover, lncRNAs have been identified as potential biomarkers and therapeutic targets for neurological diseases. Here, we give a comprehensive review of the existing knowledge of lncRNAs. Our aim is to provide a better understanding of the diversity of lncRNA structure and functions in brain development and disease. This holds promise for unravelling the complexity of neurodevelopmental and neurodegenerative disorders, paving the way for the development of novel biomarkers and therapeutic targets for improved diagnosis and treatment.

## Introduction

Over the last decades, advances in genomic sequencing of the eukaryotic transcriptome have revolutionised our perception of the complexity of human genomes. It revealed that even though most mammalian genomes are transcribed, only ~20,000 genes are encoding proteins, making <2% of the total genomic sequence, while the majority of transcripts are non-coding RNAs (ncRNAs) (ENCODE Project Consortium, 2012; Roberts et al., 2014). ncRNAs are usually classified into two groups according to the length of transcripts: small non-coding RNAs and long non-coding RNAs (lncRNAs). Small ncRNAs are less than 200 nucleotides in length, including microRNAs, Piwi-interacting RNAs, and small nuclear RNAs (snoRNAs) (Ponting et al., 2009; Nagano and Fraser, 2011). Long non-coding RNAs (lncRNAs) are more than 200 nucleotides in length that do not encode proteins and lack open reading frames (ORFs). Yet, similar to mRNA, lncRNAs are transcribed by RNA polymerase II, have similar intron/exon lengths as mRNAs, contain canonical splice sites (GU/AG), show alternative splicing patterns, can be polyadenylated or non-polyadenylated, and associate with the same types of histone modification as protein-coding genes (Roberts et al., 2014). They are also known for their secondary structure, which allows protein binding at many sites or for specific DNA–RNA binding (Wei et al., 2018). According to their genomic location, they are broadly classified into intergenic lncRNAs, intronic lncRNAs, bidirectional lncRNAs, sense lncRNAs, antisense lncRNAs, and enhancer RNAs (Ma et al., 2013; Yousefi et al., 2020).

Even though lncRNAs have no protein-coding potential, their spatiotemporal expression patterns have underlined their diverse regulatory functions (Cao et al., 2018). Many studies have shown that they play important roles in different biological processes, including regulating gene expression, both at the transcriptional and post-transcriptional level, and shaping the chromatin architecture (Lee and Bartolomei, 2013; Chen, 2016; Cao et al., 2018), in many diseases such as immunological diseases, cancer, and neurological disorders (Bian and Sun, 2011; Huarte, 2015; Wan et al., 2017).

A main challenge in molecular biology is to decode the genomic architecture that controls the function of the central nervous system (CNS). The CNS is the most complex organ in the mammalian biological system, composed of billions of neurons and glial cells that during development, differentiate from progenitor cells to mature neurons, with trillions of synaptic interactions between them (Roberts et al., 2014; Ang et al., 2020). These complex mechanisms of neuronal maturation, plasticity, and homeostasis and forming this well-orchestrated, complex cellular architecture during neurodevelopment and maintaining it during adulthood rely greatly on all the delicacies of genomic development to reach these complex cellular behaviours (Roberts et al., 2014), including the role that RNAs play in cellular regulation (Srinivas et al., 2023). lncRNAs play major roles in all phases of these processes, and therefore, it is not surprising that the CNS demonstrates the highest expression of non-coding RNA subtypes and regulatory mechanisms, with approximately 40% of all discovered lncRNAs existing in the brain (Briggs et al., 2015; Zimmer-Bensch, 2019). In this review, we summarise several known functions of lncRNAs as important genomic regulators in brain development and neurological disorders. We highlight the common functions and mechanisms of action of these transcripts. We will also discuss the latest advances in the use of lncRNAs as biomarkers and the future perspective of using lncRNAs as therapeutic targets in the treatment of neurological disorders.

## Mechanisms of lncRNAs in biological processes

### Classification of lncRNAs

Even though there are many challenges in the annotation and analysis of lncRNAs, because of the lack of a clear classification frame, the existing lncRNAs can be divided into several categories based on their function and genomic context.

**Figure 1:**
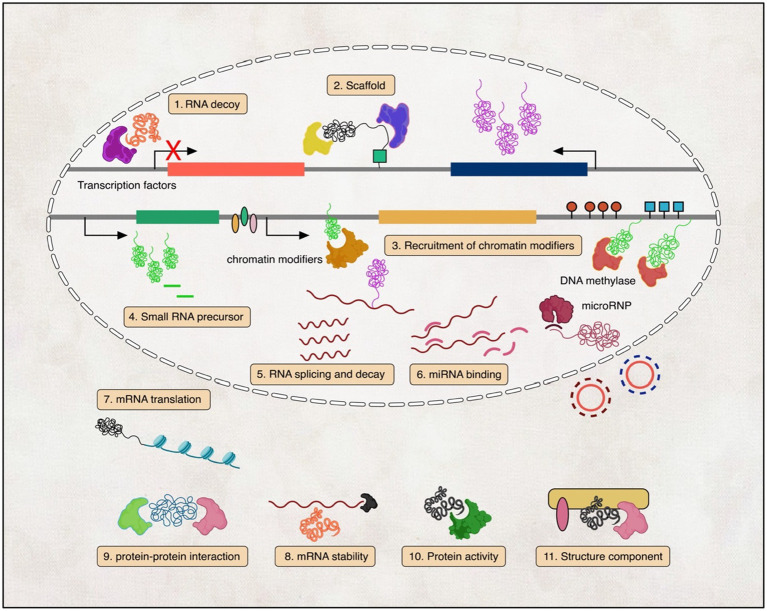
Illustration of the diverse mechanisms by which lncRNAs function. (1) lncRNAs act as transcription factor decoys; (2) lncRNAs scaffold ribonucleoprotein complexes; (3) lncRNAs recruit chromatin-modifying enzymes; (4) lncRNAs generate small regulatory RNAs; (5–8) lncRNAs regulate RNA splicing, translation, decay, and miRNA binding; and (9–11) lncRNAs engage in protein–protein interactions, regulate protein activity, and serve as structural components in the cytoplasm.

One way to categorise lncRNAs is according to their function. lncRNAs were reported to be involved in many cellular and molecular processes, like X-chromosome inactivation, imprinting, DNA methylation, transcriptional modulation, and post-transcriptional control (Mercer et al., 2009; Wilusz et al., 2009; Nagano and Fraser, 2011; Geisler and Coller, 2013; Mercer and Mattick, 2013; Zhang and Leung, 2014; Kiang et al., 2015), nuclear-cytoplasmic shuttling, translational inhibition, mRNA degradation, RNA degradation, and regulation of protein activity (Wapinski and Chang, 2011; Yoon et al., 2013; Zimmer-Bensch, 2019). Moreover, many studies have also provided evidence that lncRNAs control gene expression by interaction with DNAs, RNAs, and proteins or chromatin remodelling complexes, and more recent observations suggest that lncRNAs may in fact affect protein-coding directly (Mattick and Gagen, 2001; Khalil et al., 2009; Kiang et al., 2015; Figure 1).

Moreover, with the emergence of advanced biocomputational research tools, a large number of novel lncRNA transcripts have been identified (Amaral et al., 2011; Volders et al., 2013; Yang et al., 2013; Park C. et al., 2014; Kiang et al., 2015). These tools have also helped in predicting lncRNA interaction with various molecules genome-wide. For example, several studies have confirmed lncRNA–EZH2 interaction. EHZ2 is a member of the chromatin-modifying protein PRC2, polycomb repressive complex 2 that plays a role as tumour suppressive/oncogenic regulator. This interaction allowed the lncRNA to act as a guide for the PRC2 complex to the target site (Rinn et al., 2007; Zhao et al., 2008; Jeon and Lee, 2011). This association of lncRNAs with EZH2 is involved in the biology of tumour cells through the up- or downregulation of gene expressions (Bian et al., 2015; Zhang et al., 2015). Moreover, when we look at the downstream molecules of lncRNAs in neurological diseases, we find that they encompass a diverse range of proteins, microRNAs, and other non-coding RNAs, which participate in intricate regulatory networks. Numerous studies have highlighted specific downstream molecules associated with lncRNAs in neurological disorders. For instance, in Alzheimer’s disease (AD), the lncRNA *BACE1-AS* was found to interact with BACE1, a key enzyme involved in amyloid-β formation and AD pathogenesis (Faghihi et al., 2010). Additionally, the lncRNA *HOTAIR* was shown to modulate the expression of HOX genes by interacting with PRC2 in glioblastoma (Kciuk et al., 2023; Xin et al., 2023).

The functional diversity of lncRNAs is based on the inherent properties of RNA molecules, such as their modular organisation, ability to fold into different structures, and having many functional domains in their sequence that allow them to interact with different molecules (Wang and Chang, 2011; Zimmer-Bensch, 2019). Furthermore, compared to protein-coding genes, lncRNAs are highly tissue-specific and are usually co-expressed with neighbouring coding genes (Cabili et al., 2011). Diverse expression patterns of lncRNAs have major implications on their regulatory roles. In 2008, Mercer et al. identified hundreds of lncRNAs that are expressed in the brain by the *in situ* hybridisation method. The expression of these lncRNAs differs according to their anatomical location, cell type, and subcellular location (Mercer et al., 2008). The lncRNA *Evf2* is expressed in the ventral forebrain and was shown in an *in vivo* knockout study to regulate the development of GABAergic neurons. Other examples are the lncRNAs *MALAT1* and *Neat1*. They are both localised in the nucleus and play a role in regulating alternative splicing of pre-mRNA by modulating serine/arginine splicing factor phosphorylation (Tripathi et al., 2010). In addition, the muscle-specific long non-coding RNA, *linc-MD1*, is expressed in the cytoplasm of myoblasts and plays an important role in muscle differentiation (Cesana et al., 2011). In the next section of the review, we will elaborate more on the known mechanisms of action and functional roles of lncRNAs.

**Figure 2:**
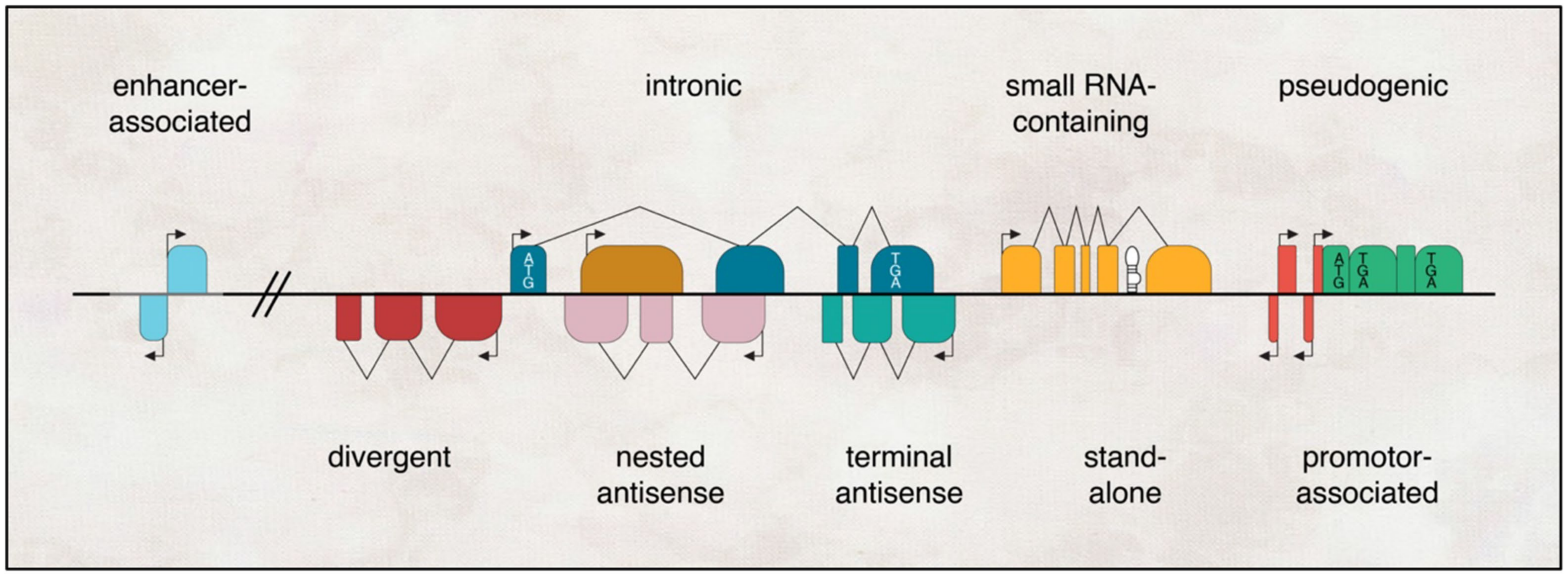
Genomic landscapes of lncRNAs encompass diverse contexts. They can exist as independent transcriptional units that arise from enhancers, promoters, or introns of other genes (where the protein-coding gene is depicted with a white box denoting the start codon ATG and stop codon TGA); originate from pseudogenes (marked with a premature stop codon TGA in black); or emerge as antisense transcripts to other genes, exhibiting varying degrees of overlap, ranging from none (divergent) to partial (terminal) to complete (nested). Additionally, lncRNAs can serve as hosts for one or more small RNAs (represented by black hairpins) within their transcriptional units.

Another way to categorise lncRNAs is according to their genomic location, as in, from where in the genome they are being transcribed. These lncRNAs can be classified into five different groups: Stand-alone lncRNAs, which are transcribed from a specific sequence that does not overlap with a protein-coding gene; antisense transcripts, transcribed opposite to the sense DNA sequence; Pseudogenes, transcribed from genes that lost their coding potential due to a mutation; Intronic, transcribed from an intron sequence; Intergenic, lncRNAs that are promoter-associated and enhancer associated transcripts (Mercer et al., 2009; Kung et al., 2013; Figure 2). It is important to mention that this genomic context categorisation does not provide any information about their function or conservation. In addition, studies showed that the majority of lncRNAs are actually localised in the cytoplasm, instead of the nucleus and associated with ribosomes, where they may help in the evolution of new protein subtypes (Ruiz-Orera et al., 2014; van Heesch et al., 2014).

### Mechanisms of lncRNA action

**Figure 3:**
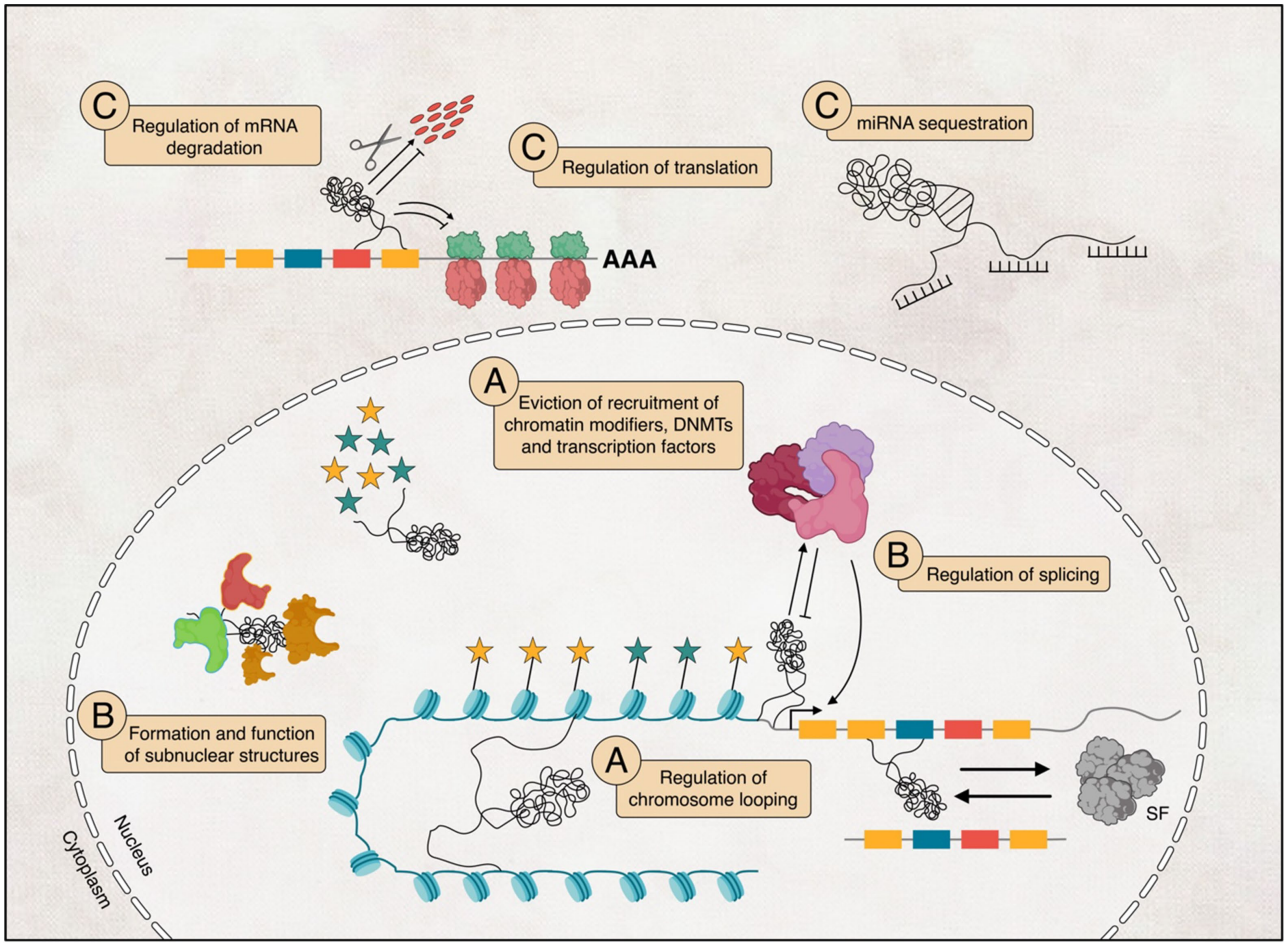
The potential functional diversity of long non-coding RNAs (lncRNAs) in their involvement in regulating transcription (A), influencing post-transcriptional processes within the nucleus (B), and their potential implications in interfering with translation (C) within the cytoplasm.

Even though we do not understand the full functions of lncRNAs, many studies have shown that they play various roles in almost every aspect of biological regulations, from chromatin structure to protein level (Wilusz et al., 2009; Wu et al., 2013). Here, we summarise lncRNAs’ broad mechanisms in regulating gene expression, including chromatin modification, transcription, and post-transcription regulations (Figure 3).

#### LncRNAs function in transcriptional regulation

lncRNAs can regulate the transcription of target genes by different mechanisms. They can act either in *cis* or *trans* to regulate the transcription of local or distal genes (Wilusz et al., 2009; Zimmer-Bensch, 2019). Due to their secondary structure, they can act as a scaffold to recruit chromatin-modifying complexes, transcription factors (TFs), and DNA methyltransferases to specific genomic locations (Batista and Chang, 2013; Zimmer-Bensch, 2019). These lncRNA-formed complexes regulate target genes by either activating or repressing their expression (Marchese et al., 2017; Zimmer-Bensch, 2019).

Many lncRNAs have been described to modify chromatin structure by recruiting chromatin remodelling factors such as histone H3K4 methyltransferases, which catalyses the trimethylation at histone 4 lysine 3 residues and activate the transcription of target genes (Wang et al., 2011; Cabianca et al., 2012; Zimmer-Bensch, 2019), and Polycomb Repressive Complex 2 (PRC2) that catalyses the trimethylation of H3K27 residues that leads to chromatin condensation and silencing of target genes (Margueron and Reinberg, 2011; Zimmer-Bensch, 2019). The lncRNA Hox Transcript Antisense RNA (*HOTAIR*) is expressed across the HOXC locus and is known to act in trans to recruit PRC2 to modify the chromatin and repress the transcription of HOXD locus, which is 40 kb away from its site of synthesis (Rinn et al., 2007). Another prominent example is the role that the lncRNA *Xist* plays in X-chromosome inactivation. It has been shown that the lncRNA *Xist* interacts with another lncRNA, the lncRNA *RepA*, which was found to be both part of the *Xist* lncRNA as well as expressed by itself (Zhao et al., 2008; van de Vondervoort et al., 2013). The *RepA* lncRNA can bind the histone methyltransferase enhancer of Zester Homolog 2 (Ezh2), a subunit of the PRC2. The lncRNA *Xist* recruits the PRC2 complex through the *RepA* sequence, allows trimethylation on lysine-27 of H3 histones (H3K27), and efficiently modifies the chromatin, repressing gene expression, to inactivate the X-chromosome (Zhao et al., 2008; van de Vondervoort et al., 2013).

In addition to their role in recruiting histone-modifying complexes, lncRNAs can interact with DNA-/RNA-binding proteins, including DNA methyltransferases like DNMT1 and DNMT3b and TFs, preventing or promoting their recruitment to the DNA to repress or activate target genes (Marchese et al., 2017; Zimmer-Bensch, 2019). The lncRNA *Dali*, a conserved central nervous system expressing intergenic lncRNA, binds to DNMT1 and controls in trans the DNA methylation status of CpG island-associated promoters to promote neuronal differentiation (Chalei et al., 2014; Zimmer-Bensch, 2019). Another example is the lncRNA *Evf2*, which is transcribed from Dlx-5/6 enhancer and acts as a co-activator by recruiting the TF Dlx-2 to Dlx-5/6 gene and inducing its expression (Feng et al., 2006).

#### LncRNAs function in post-transcriptional regulation

Apart from their role in transcriptional control, lncRNAs are involved in post-transcriptional regulation by acting as antisense transcripts to regulate RNA processing, including alternative splicing, mRNA stability, nuclear-cytoplasmic shuttling, and translation (Zhang et al., 2013; Ariel et al., 2014; Bardou et al., 2014; Romero-Barrios et al., 2018; Zimmer-Bensch, 2019).

#### Alternative splicing

It has been shown that several brain-expressed nuclear-localised lncRNAs were involved in pre-mRNA splicing and alternative splicing, e.g., the lncRNAs *NEAT1* and *MALAT1* (Qureshi and Mehler, 2012; Briggs et al., 2015; Romero-Barrios et al., 2018).

These lncRNAs can recognise splicing factors and control their posttranslational modifications, such as phosphorylation, or regulate their interaction with other splicing factors. Moreover, lncRNAs can mediate alternative splicing through chromatin remodelling (Romero-Barrios et al., 2018). The process of splicing requires rounds of phosphorylation and dephosphorylation of serine/arginine (SR) protein domains that will allow the binding of these proteins to the target pre-mRNA to influence their splicing (Cao et al., 1997; Xiao and Manley, 1997, 1998).

The lncRNAs *NEAT1* and *MALAT1* were shown to bind with CLK kinase and control the phosphorylation of splicing factors. *NEAT1* regulates the phosphorylation of SRp40, which is involved in the processing of the PPARy pre-mRNA into the PARy2 mRNA (Cooper et al., 2014). While *MALAT1* regulates the phosphorylation of SR of MALAT1-interacting SRSF1 protein in the nucleus (Tripathi et al., 2010). In contrast, dephosphorylation of SRSF1 controls the export of mRNA-associated proteins and assists in binding with cytoplasmic mRNAs to effect translation (Huang et al., 2004; Sanford et al., 2005).

### mRNA stability

Many lncRNAs that were discovered play a role in mRNA stability in the cytoplasm. The lncRNA *BACE1-AS*, antisense transcript for β-secretase 1, promotes mRNA stability (Faghihi et al., 2008; Kretz, 2013). While the lncRNA GADD7, growth-arrested DNA-damage inducible gene 7, decreases the stability of mRNAs (Gong and Maquat, 2011; Liu et al., 2012).

#### Translation

Gene regulation at a translational level is important in many processes, including neuronal function. LncRNAs are shown to play important roles in either promoting or repressing translation through different mechanisms. The antisense lncRNA *AS-Uchl1* recruits the Uchl1 mRNA to polysomes, thus promoting translation (Carrieri et al., 2012), while the *lincRNA-p21* suppresses the translation of target transcripts by enhancing ribosome drop-off (Yoon et al., 2012). Moreover, lncRNAs can affect translation by competing for miRNA binding. They contain multiple miRNA-binding sites, and through binding to these miRNAs, they prevent their binding to coding mRNAs and so stop miRNA-dependent effects on translation (Franco-Zorrilla et al., 2007; Karreth et al., 2011; Salmena et al., 2011; Tay et al., 2011; Chen et al., 2015; Peng et al., 2015).

### Functionality of lncRNAs

Even though lncRNAs are extremely abundant, they were initially classified as transcriptional noise or “junk” DNA. The low sequence conservation and low transcription potential of lncRNAs fuelled the debate about their function and suggested low evolution pressure and biological importance (Mercer et al., 2009; Ponting et al., 2009; Ponting and Belgard, 2010). One study argued that lncRNAs are transcriptional noise and showed that most of the lncRNA transcripts discovered in their sequencing analysis are associated with known genes (van Bakel et al., 2010; Roberts et al., 2014). However, this hypothesis was opposed by other studies that suggested this association between lncRNAs and coding protein loci is consistent with persistent transcription and suggests inadequate sequencing depth in the opposing study (Clark et al., 2011). Moreover, a different finding reported by the GENCODE consortium suggests that most lncRNAs are actually expressed as independent transcripts (Derrien et al., 2012). Nevertheless, many research groups studied lncRNAs and confirmed the wide-ranging functional roles of lncRNAs.

First, lncRNAs are expressed in a tissue-specific manner. Analysing deep sequencing data to understand the transcriptional landscape of different cell lines shows that 29% of lncRNAs were expressed in a cell-specific manner, while only 10% were expressed in all cell types, in contrast to protein-coding genes for which the expression percent were 7 and 53%, respectively (Djebali et al., 2012). Interestingly, 40% of all lncRNAs are expressed in the brain (Derrien et al., 2012).

Moreover, genome-wide expression profiling of lncRNAs in the brain has been carried out using different methods. The Allen Brain Atlas (ABA) is a large study that maps the expression of genes in the developing and adult mouse brain at a genome-wide scale. The ABA used RNA *in situ* hybridisation (ISH) to visualise the expression of 849 ncRNAs that are expressed in the adult brain and found that most lncRNAs are associated with specific neuroanatomical loci. For example, the lncRNA *AK037594* was found to be expressed only in the dentate gyrus and CA1–3 regions of the hippocampus. While *Gomafu* is expressed only in differentiating neural progenitors and a subset of postmitotic neurons (Sone et al., 2007).

Interestingly, most of the lncRNAs that were identified to be highly expressed in the brain are found to be binding to TFs and involved in the transcription regulation of nearby protein-coding genes that are involved in brain development (Ponjavic et al., 2009; Augoff et al., 2012), which suggests that they have explicit biological role. For example, the ABA shows expression of the lncRNA *Evf2*, which interacts in trans with the TF Dlx-2 to regulate the expression of the Dlx-6 gene (Feng et al., 2006). Consistent with this function, *Evf2* shows a coincident expression profile with Dlx-2, which supports its role in neuronal differentiation (Feng et al., 2006).

Second, lncRNAs showed signs of regulated expression (Ravasi et al., 2006). For example, 174 lncRNAs were differentially expressed during the *in vitro* 16 days of differentiation of mouse embryonic stem (ES) cells into embryoid bodies (Dinger et al., 2008). Another study showed different expression patterns of four lncRNAs after treatment with retinoic acid to induce neuronal differentiation in mouse ES cells (Sheik Mohamed et al., 2010). Moreover, pluripotency factors, such as Oct4 and Nanog, bind to the promoters of some lncRNA and control their transcription. This suggests that they play a role in the balance between maintenance of pluripotency and lineage commitment. Knockdown and overexpression of these lncRNAs affected the expression of Nanog and Oct4 and promoted lineage-specific differentiation (Sheik Mohamed et al., 2010).

Third, unlike protein-coding genes, which are highly conserved and must preserve their ORF, lncRNAs can only have shorter stretches of sequence that are conserved to sustain their functional domain and structure (Pang et al., 2006). For example, the lncRNA *Xist* is known for its function in X-chromosome inactivation, but only a short sequence of its length is conserved, and despite this, PRC2 targeting region in Xist is defined in different species. Functionality and high conservation of the sequence may not always correspond to each other (Hendrich et al., 1993; Nesterova et al., 2001; Zhao et al., 2008).

## Function of lncRNAs in neurogenesis

The central nervous system is considered the most complex organ with the most elaborate biological system in the mammalian body. Understanding the molecular mechanisms underlying the function of this organ is a big challenge and is considered as a subject of interest among many scientists. In the mammalian brain, neurogenesis is a dynamic process associated with NSC and NPC differentiation into newborn neurons that integrate into the local neural network (Mattick, 2007; Vieira et al., 2019). This process is governed by a complex biological and molecular system that regulates NSC proliferation and differentiation in postnatal and adult brain development, which takes place in the SVZ and SGZ in the DG of the hippocampus (Ming and Song, 2011; Yao and Jin, 2014; Ayana et al., 2017).

lncRNAs associated with neural genes display positive selection and rapid evolution, which suggests new insights to link genetics to the evolution of the human brain. Due to the diverse biological roles of lncRNAs that accord with the complexity of the brain, lncRNAs were suggested as ideal candidates and emerged as important epigenetic regulators in controlling neural development, proliferation, and differentiation, including cell line restriction, cell fate determination, and continuing stage differentiation (Mattick, 2007; St Laurent and Wahlestedt, 2007). Therefore, recent studies have started to investigate the neurobiological roles of lncRNAs in the brain (Mercer et al., 2008; Ponjavic et al., 2009; Belgard et al., 2011). In this section, we delve into the regulatory role of lncRNAs in neurogenesis, providing a comprehensive overview that is summarised in Table 1.

**Table 1:** The role of lncRNAs on neurogenesis.

lncRNA name	Mechanism	Biological function	References
BRN1B	Controls proliferation of progenitors in the SVZ of the developing cortex.	Organ and brain development	Sauvageau et al. (2013)
HAR1F (HAR1A)	Specifically expressed in Cajal–Retzius neurons	Cortical neuron specification and migration	Pollard et al. (2006)
Evx1as and Hox5b/6as	Specifically associated with Dlx-family genes	Brain development in mammals and Drosophila	Dinger et al. (2008)
Evf2	Interacts with Dlx-2 to regulate transcriptional activity of Dlx-5/6 and Gad1	Regulates GABAergic interneurons formation	Berghoff et al. (2013), Bond et al. (2009), and Cajigas et al. (2018)
Trincr	Regulates ERK signalling pathway	Restrains fibroblast growth factors and suppresses NPC self-renewal	Lanner and Rossant (2010) and Li Y. P. et al. (2019)
LincRNA-ROR	Forms a regulatory feedback loop with miR-145 and Oct4, Sox2, and Nanog	Enhances reprogramming of iPSCs and regulates ESC pluripotency	Loewer et al. (2010) and Wang et al. (2013)
MIAT	Functions as a co-activator of Oct4	Regulates ESC proliferation	Sheik Mohamed et al. (2010)
Sox2OT	Modulate Sox2 gene	Maintains self-renewal of neural stem cells	Fantes et al. (2003)
ZNF281	Controls NF-κB1 signalling pathway	Regulates self-renewal and proliferation in GSCs	Katsushima et al. (2016), Li X. T. et al. (2019), and Tang et al. (2019)
TALNEC2	Control genes important for the growth, stemness, and mesenchymal transformation of GSCs such as Nanog, SOX2 and Oct4, and CTGF	Regulates self-renewal and pluripotency in GSCs	Brodie et al. (2021)
Linc01198	Function as a scaffold to recruit and enhance NEDD4-1-dependent repression of PTEN expression	Regulates self-renewal and pluripotency in GSCs	Chen W. L. et al. (2019)
Nkx2.2AS	Regulates mRNA level of Nkx2.2 TF	Regulates oligodendrocyte differentiation	Tochitani and Hayashizaki (2008)
Six3OS	Controls the function of the TF Six3 by acting as a molecular scaffold to recruit histone modification enzymes to Six3 gene	Regulates retinal cell specification	Rapicavoli et al. (2011)
Dlx1AS	Regulates expression of neighboring homeobox genes	Modulate GABAergic neurons and oligodendrocyte differentiation	Mercer et al. (2010) and Ramos et al. (2013)
HOTAIRM1	Regulator of HOXA genes	Controls neuronal differentiation	Lin et al. (2011)
Pnky	Forms a complex with splicing factor and PTBP1 to control NSCs differentiation to neurons through alternative splicing	Controls NSC differentiation to neurons	Grammatikakis and Gorospe (2016) and Ramos et al. (2015)
LncR492	Binds with mRNA-binding protein HuR and activates Wnt signalling	Inhibits neuronal differentiation	Winzi et al. (2018)
BDNF-AS	Suppresses neurite growth through activation of TrkB signalling pathway	Controls neuronal differentiation	Zheng et al. (2016)
Sox1ot	Regulates expression of Sox1 TF	Maintain the stemness of NSCs	Ahmad et al. (2017), Askarian-Amiri et al. (2014), Kan et al. (2004), and Knauss et al. (2018)
Sox2ot	Regulates expression of Sox2 TF	Maintain the stemness of NSCs	Ahmad et al. (2017), Askarian-Amiri et al. (2014), Kan et al. (2004), and Knauss et al. (2018)
RMST	Allows Sox2 binding to target genes	Controls neurogenesis	Ng et al. (2013a)
Kdm2b	Binds to Kdm2b gene and increases its expression	Regulates neuronal differentiation	Li et al. (2020)
Paupar	Forms RNA multiprotein complex with Pax6 and KAP1 TFs	Controls neural differentiation	Pavlaki et al. (2018) and Vance et al. (2014)
Six3OS	Functions as a molecular scaffold to regulate Six3 activity	Controls eye development and postnatal retinal cell specification	Rapicavoli et al. (2011)
LncRNA 1604	Regulates miR-200c to control key TFs ZEB1/2	Controls neural differentiation	Weng et al. (2018)
Rik-201	Acts as a ceRNA of miR-96 to controls Sox6 gene.	Controls neural differentiation	Zhang et al. (2019)
Rik-203	Acts as a ceRNA of miR-467a-3p to control Sox6 gene.	Controls neural differentiation	Zhang et al. (2019)
MEG3	Acts as a negative regulator of miR-128-3p	Enhances neuronal differentiation	Gao et al. (2019)
Malat1	1. Regulates MAPK, PPAR and p53 signalling pathways 2. Regulates the expression of Nlgn1 and SynCAM1 synaptogenesis genes.	1. Controls neurite growth occurring in early neuronal differentiation stage 2. Controls synaptic function and dendrite development	Chen et al. (2016) and Bernard et al. (2010)
LncRNA-AK053922	Regulates sonic hedgehog (Shh) signalling	Controls neural cell type specification	Hashimoto-Torii et al. (2003), Meyer and Roelink (2003), and Wu et al. (2013)
NOS pseudogene antisense transcript	Controls expression of NOSs mRNAs	Supports synaptic strength and memory formation	Kemenes et al. (2002) and Korneev et al. (1999)
BC1	Controls protein synthesis in postsynaptic dendritic microdomains	Maintenance of synaptic plasticity	Muddashetty et al. (2002)
BC200	Controls protein synthesis in postsynaptic dendritic microdomains	Maintenance of synaptic plasticity	Muddashetty et al. (2002)
BDNF-AS	1. Interacts with BDNF mRNA and controls BDNF gene function 2. Interacts and recruits EZH2 subunit of PRC2 to BDNF gene promotor to inhibit its expression	Controls synaptic plasticity and memory formation	Lipovich et al. (2012), Modarresi et al. (2012), and Pruunsild et al. (2007)

### LncRNAs and brain development

Scientists have used recent high-throughput technologies like sequencing, microarray expression, and high-throughput RNA *in situ* hybridisation method (Allen Brain Atlas (ABA) study) to visualise the expression of over 20,000 transcripts at cellular resolution (Lein et al., 2007). They observed thousands of lncRNAs expressed in the brain during neural development. Utilising these data for more analysis, we found that 849 lncRNAs examined out of 1,328 exhibit specific expression profiles in distinct neuroanatomical regions, cell subtypes, and subcellular compartments in different adult mouse brain regions (Mercer et al., 2008; Ng et al., 2013b; Shi et al., 2017). In the mice model, lncRNAs showed a different expression pattern across the cortical layers in addition to being specifically expressed in the dentate gyrus, subventricular zone, and olfactory bulb regions of the brain (Belgard et al., 2011; Ramos et al., 2013). In human neocortical brain sections, lncRNAs were shown to be expressed in an age-dependent pattern from infancy to adulthood (Lipovich et al., 2014). This was confirmed in a study done *in vivo* in mouse of evolutionarily conserved intergenic lncRNAs, in which scientists identified “brain clusters” of lncRNAs (Guttman et al., 2009) that are differentially expressed during development (Mercer et al., 2010). Moreover, in a functional study by Sauvageau et al. (2013) they showed that lncRNAs, in particular *BRN1B*, are important for organ and brain development using several lncRNA *in vivo* mice knockout models (Sauvageau et al., 2013). While HAR1, one of the most evolutionary acceleration regions in the human genome, was discovered to belong to a lncRNA gene, *HAR1F (HAR1A)*, which was shown to be specifically expressed in Cajal–Retzius neurons in the developing human neocortex during the period for cortical neuron specification and migration (Pollard et al., 2006). Another example is the lncRNAs *Evx1as* and *Hox5b/6as*, which were shown to be specifically associated with genes from the *Dlx* family that are known to be involved in brain development in mammals and Drosophila (Dinger et al., 2008). In addition, embryonic ventral forebrain-2 (*Evf2*) is transcribed antisense to the *Dlx-6* gene, which is a homeobox-containing TF important in forebrain neurogenesis (Stenman et al., 2003; Feng et al., 2006).

Interestingly, a breakthrough study in *in vivo* samples obtained surgically from human neocortical tissue by Lipovich et al. (2014) identified lncRNA expression in different ages and found eight lncRNAs with strong statistical associations with ageing and, by so, brain development. Most of these lncRNAs were transcribed antisense from neighbouring protein-coding genes that are known to play a role in neural development. This implies that lncRNAs and protein-coding genes interact and play functional regulatory mechanisms in neural development (Mercer et al., 2010). Importantly, these lncRNAs contain specific anthropoid exons and mRNA processing regions that reside within primate-specific sequences, which confirms their recent evolutionary origins (Lipovich et al., 2014). Furthermore, gene expression analysis studies of the mouse retina at different time points discovered many evolutionary conserved lncRNAs that are expressed in the developing retinal cells, which further confirms that lncRNAs play a functional role in neuron development (Blackshaw et al., 2004). Altogether, these findings suggest the involvement of lncRNAs in the development of the human brain.

### LncRNAs and NSC self-renewal and proliferation

Neural stem cells (NSCs) have a significant ability for self-renewal and proliferation, which are important for CNS homeostasis (Hosseinkhani et al., 2013; Zhao et al., 2020). Although the underling regulatory mechanism is still not clear, recent studies confirmed that lncRNA functions as a modulator in NSC self-renewal and proliferation (Zhao et al., 2020). For example, overexpression of TRIM71 interacting long non-coding RNA 1, *Trincr*, regulated kinase (ERK) signalling pathway, which is essential for cell self-renewal, restrains fibroblast growth factors (FGF) and therefore suppresses NPC self-renewal (Lanner and Rossant, 2010; Li Y. P. et al., 2019). In addition, the long intergenic non-protein-coding RNA regulator of reprogramming, *lincRNA-ROR*, was shown to enhance the reprogramming of induced pluripotent stem cells (iPSCs) and regulate the maintenance of embryonic stem cell (ESC) pluripotency through forming a regulatory feedback loop with miR-145, Oct4, Sox2, and Nanog (Loewer et al., 2010; Wang et al., 2013). Similar to this is the lncRNA *MIAT*, myocardial infarction-associated transcript, which functions as a co-activator of Oct4. Loss of *MIAT* inhibits the expression of Oct4, Sox2, and Klf4 and reduces ESC proliferation (Sheik Mohamed et al., 2010). An interesting example is the Sox2 Overlapping Transcript, *Sox2OT*, a highly conserved lncRNA that overlaps the Sox2 gene (Fantes et al., 2003). Sox2 is a TF that is important in maintaining self-renewal of neural stem cells (Mizuseki et al., 1998). *Sox2OT* was shown to be expressed in neural stem cells and is downregulated during differentiation (Amaral et al., 2009).

Furthermore, glioblastoma stem-like cells (GSCs) exhibit the stemness properties of stem cells, like the ability to self-renew and multipotency (Cheng et al., 2013), which were shown to be regulated by lncRNAs. The lncRNA *ZNF281* is a newly identified lncRNA that participates in controlling self-renewal and proliferation in GSCs through targeting NF-κB1 signalling pathway (Katsushima et al., 2016; Li X. T. et al., 2019; Tang et al., 2019). Moreover, the lncRNA *TALNEC2*, tumour-associated lncRNA expressed in chromosome 2, and *linc01198* were also found to regulate self-renewal and pluripotency of GSCs (Chen W. L. et al., 2019; Brodie et al., 2021). Thus, lncRNA may become novel potential therapeutic target for glioblastoma therapy.

### LncRNAs and NSC differentiation

lncRNAs also play roles in neural cell fate determination, neuronal-glia fate differentiation, and oligodendrocyte expansion. In validation of the above studies, identified lncRNAs using microarray expression analysis in mouse cell lines. They discovered that lncRNAs are differentially expressed between embryonic forebrain-derived neural stem cells (NSCs), GABAergic neuron/oligodendrocyte cells, and the different stages of differentiated neurons and glia. For example, the lncRNA *Malat1*, metastasis-associated lung adenocarcinoma transcript 1, was downregulated in precursor cells but upregulated in differentiated neuronal and glial cells. When precursor cells were treated with histone deacetylase (HDAC) inhibitor, which is known to suppress the maturation of oligodendrocyte precursor cells and convert them to neuronal pattern, the expression of lncRNAs was also affected, suggesting that they are being regulated by HDAC (Mercer et al., 2010). Another study observed the function of the lncRNA *Nkx2.2AS*, a natural antisense transcript overlapping the TF gene Nkx2.2, which was shown to regulate oligodendrocyte differentiation. Overexpression of Nkx2.2AS induced oligodendrocyte differentiation and increased Nkx2.2 mRNA expression (Tochitani and Hayashizaki, 2008). In addition, the lncRNA *Six3OS*, which is transcribed from the opposite strand of gene encoding the homeodomain TF Six3. *Six3OS* controls Six3 function by acting as a molecular scaffold to recruit histone modification enzymes to the Six3 gene, which results in regulating retinal cell specification (Rapicavoli et al., 2011). Another example, *Dlx1AS* lncRNA, the antisense transcript of the distal-less homeobox 1 (Dlx-1), was shown to be upregulated during GABAergic differentiation and downregulated during oligodendrocyte differentiation (Mercer et al., 2010). *Dlx1AS* functions in neuronal differentiation by regulating expression of neighbouring homeobox genes (Ramos et al., 2013). Moreover, the lncRNA *Evf2*, which regulates GABAergic interneurons formation in the developing mouse and human brain through controlling the expression of Dlx-5, Dlx-6, and glutamate decarboxylase 1 (Gad1), an enzyme responsible for catalysing glutamate to form GABA (Bond et al., 2009; Berghoff et al., 2013; Cajigas et al., 2018). Interestingly, RNA-seq analysis of the expression of lncRNAs in human neurons derived from iPSC found that these lncRNAs dramatically changed during the transition from iPSC to differentiated neurons. Like the lncRNA *HOTAIRM1*, which is known to be a regulator of several HOXA genes during myelopoiesis, it was shown to be upregulated in differentiated neurons (Lin et al., 2011).

### The role of lncRNAs on repressing neuronal differentiation

Unlike the above-mentioned examples of lncRNAs that are highly expressed in the brain and known to promote neuronal differentiation, some other lncRNAs were shown to be downregulated in the brain and repress neuronal differentiation. For example, the lncRNA *Pnky* is known for its role in inhibition of neuronal development, and its expression was decreased during V-SVZ NSC differentiation into neuronal cells. It forms a complex with splicing factor and RNA-binding protein (RBP)-polypyrimidine tract-binding protein (PTBP1) and functions in controlling NSC differentiation in neurons through alternative splicing. Knockdown of *Pnky* or PTBP1 promoted neurogenesis in cultured postnatal V-SVZ NSC differentiation processes in mature neurons (Ramos et al., 2015; Grammatikakis and Gorospe, 2016). Moreover, the lncRNA *lncR492* functions as an inhibitor of neuronal differentiation by binding with mRNA-binding protein HuR and activation of Wnt signalling (Winzi et al., 2018). Additionally, the lncRNA *BDNF-AS*, brain-derived neurotrophic factor antisense, was shown to control neuronal differentiation. Its overexpression suppressed neurite growth in ketamine-treated mouse embryonic NSC-derived neurons through activation of the potassium uptake system protein (TrkB) signalling pathway (Zheng et al., 2016).

### LncRNAs effect on neighbouring genes expression and binding to transcription factors

LncRNAs can control neural development by controlling the expression of proximal protein-coding genes. For example, Sox1 and Sox2 are TFs known to maintain the stemness of NSCs. Recently, the lncRNA *Sox1ot*, Sox1 overlapping transcript, and lncRNA *Sox2ot*, Sox2 overlapping transcript, were discovered. They are evolutionarily conserved lncRNAs that are highly expressed during neural development and overlap with Sox1 and Sox2 expression, respectively (Kan et al., 2004; Askarian-Amiri et al., 2014; Ahmad et al., 2017; Knauss et al., 2018). The lncRNA *Sox2ot* suppressed NSC proliferation and neuronal differentiation by associating with the transcriptional factor YY1, which binds to CpG island in the Sox2 locus to suppress Sox2 expression (Knauss et al., 2018).

Another example is the lncRNA *RMST*, rhabdomyosarcoma 2-associated transcript, which has been shown to be important in neurogenesis. *RMST* allowed Sox2 binding in the promoter of target genes, and its knockdown led to differential expression of almost 1,000 genes important in neurogenesis (Ng et al., 2013a). Moreover, the lncRNA *Kdm2b* binds to the Kdm2b gene and increases its expression, which promotes neuronal differentiation in cortical projection neurons (Li et al., 2020). Similarly, the lncRNA *Paupar* is a CNS-expressed and chromatin-associated lncRNA that is transcribed upstream of the gene encoding the Pax6 TF. It plays a role in neural differentiation by forming RNA multiprotein complex with Pax6 and KAP1 TFs (Vance et al., 2014; Pavlaki et al., 2018). Furthermore, in mammalian eye development, the retina consists of cell-specific subtype neuron layers connected by synapses (Ng et al., 2013b). *Six3OS* is the long non-coding opposite strand transcript (lncOST) of the homeodomain factor Six3. It controls eye development and postnatal retinal cell specification through its function as a molecular scaffold to regulate Six3 activity (Rapicavoli et al., 2011).

LncRNAs also bind to TFs to regulate neurogenesis, like the TFs SUZ12 (a component of the polycomb repressive complex 2, PRC2), REST, and SOX2 (a pluripotency-associated TFs), in which they act as guides for these proteins. REST is a TF that is known to regulate pluripotency and control neurogenesis. It represses the expression of genes involved in neurogenesis in non-cells (Chong et al., 1995). A study by Johnson et al. discovered two lncRNAs that are regulated by REST in NSCs (Johnson et al., 2009). Similarly, the transcription co-factor CoREST, which also functions in repressing neural genes (Andres et al., 1999). CoREST was identified to associate with 63 lncRNAs in RIP-chip assay; most of them also bind to PRC2, implying that these lnRNAs may also function in regulating neural cell differentiation (Khalil et al., 2009). In addition, Dlx genes encode homeodomain proteins that are known for their function in controlling neuronal differentiation and migration (Anderson et al., 1997a,b). As mentioned above, the lncRNA *Evf2* is transcribed from the Dlx locus and interacts with Dlx-4 protein to increase its transcriptional activation functionality in NSCs (Feng et al., 2006). *Evf2* knockout mice model showed an abnormal gene expression pattern that led to a decrease in the number of GABAergic interneurons in the mouse hippocampus (Bond et al., 2009).

### LncRNAs acting as ceRNA of miRNA

miRNAs are short non-coding RNAs (approximately 22 nucleotides in length) that are expressed abundantly during brain development and are known to suppress the translation of coding genes in all stages of neural differentiation (Shi et al., 2010). LncRNAs were shown to function as competing endogenous RNAs (ceRNA) and control miRNA to regulate genes important for neural development (Tay et al., 2014; Weng et al., 2018). For example, knockdown of LncRNA *1,604* suppressed neural differentiation by regulating miR-200c to control key TFs zinc finger E-box binding homeobox 1/2 (ZEB1/2) (Weng et al., 2018).

Moreover, lncRNA could be processed to generate several variants that play roles in neurogenesis. The lncRNA C130071C03 Riken variants, *Rik-201* and *Rik-203*, are activated by neurogenesis TF CCAAT/enhancer-binding protein β (C/EBPβ) and therefore modulate brain development. Knockdown of *Rik-201* and *Rik-203* suppressed the expression of neural differentiation-related gene Sox6, and therefore repressed neural differentiation by function as ceRNAs of miR-96 and miR-467a-3p, respectively (Zhang et al., 2019). Additionally, miR-128-3p is highly expressed in the brain and controls neural differentiation. Overexpression of miR-128-3p suppressed neurons but enhanced gliocyte differentiation. In addition, the LncRNA *MEG3* is elevated by the cAMP/response element-binding protein (CREB) pathway. Thus, it enhances neuronal differentiation by acting as a negative regulator of miR-128-3p (Gao et al., 2019).

### Emerging as key signalling pathway modulators

LncRNAs could also contribute to neural differentiation by being affected by signalling pathway. Neurite outgrowth occurs in the early neuronal differentiation stage. The lncRNA metastasis-associated lung adenocarcinoma transcript 1 (*Malat1*) was found to play a crucial role in neurite growth. Knockdown of *Malat1* prevents neurite outgrowth and advanced cell death by suppressing the mitogen-activated protein kinase (MAPK) signalling pathway and stimulating the peroxisome proliferator-activated receptor (PPAR) and p53 signalling pathways (Chen et al., 2016). Another example is lncRNA-*AK053922*, which has been shown to control neural cell type specification through regulating sonic hedgehog (Shh) signalling (Hashimoto-Torii et al., 2003; Meyer and Roelink, 2003; Wu et al., 2013).

### LncRNAs in synaptic plasticity, cognitive function, and memory

Synaptogenesis is a critical process during neuronal development, which is altered in many neurodevelopmental disorders (Zoghbi, 2003; Ecker et al., 2013). LncRNAs have been shown to play important and direct roles in regulating genes involved in synaptic plasticity, cognitive function, and memory. GABAergic inhibitory interneurons in the hippocampus are responsible for learning in the embryonic and adult brains. Studies showed that the lncRNA *Evf2*, which is transcribed from the Dlx-5/6 ultraconserved region, is important for the development of GABAergic neurons. It interacts with the transcription co-activator Dlx-2 to regulate the transcriptional activity of Dlx-5/6 and glutamate decarboxylase 1 (Gad1, necessary for the conversion of glutamate to GABA) (Feng et al., 2006), and then controls the expression of genes that regulate GABAergic interneurons in the developing mouse brain. Knockdown of *Evf2* causes abnormal formation of GABAergic circuitry in the hippocampus and dentate gyrus, which affects synaptic activity in mice (Bond et al., 2009). Moreover, gene ontology analysis revealed that genes affected by the lncRNA *Malat1* were mostly associated with synaptic function and dendrite development. Knockdown of *Malat1 in vitro* in primary hippocampal neurons decreased synaptic density and changed the expression of Nlgn1 and SynCAM1 genes that are known to regulate synaptogenesis (Bernard et al., 2010).

LncRNAs also function to support long-term changes in synaptic strength. Nitric oxide (NO) is a signalling molecule that functions as a neurotransmitter and thus is important in learning, long-term potentiation (LTP), and long-term depression (LTD) (Muller, 1996). Nitric oxide synthases (NOSs) are enzymes that function to catalyse the production of NO from l-arginine. Interesting research done in *Lymnaea stagnalis* snail discovered that an antisense RNA is transcribed from the NOS pseudogene and complements the NOS mRNA. Reduction of NOS pseudo-gene antisense transcript causes upregulation of NOS mRNA levels transiently, and the timing overlapped with the window for memory formation, which suggests that the antisense NOS pseudogene transcripts associate with memory formation by controlling the expression of NOS mRNAs (Korneev et al., 1999; Kemenes et al., 2002).

The rodent-specific BC1 and the non-homologous primate-specific BC200 lncRNAs function to control protein synthesis in postsynaptic dendritic microdomains; therefore, they play important roles in maintenance of synaptic plasticity (Muddashetty et al., 2002). Neurogranin (Nrgn) and calcium/calmodulin-dependent protein kinase II inhibitor 1 (Camk2n1, CaMKIINalpha) are proteins that are expressed in rodents’ brains and have been shown to regulate synaptic long-term potentiation by controlling Ca^2+^/calmodulin-dependent protein kinase II (CaMKII) (Gerendasy and Sutcliffe, 1997; Kennedy, 1998; Lisman et al., 2002). It has been found that transcripts that encode the sense and antisense of the gene locus of these two proteins control their post-transcriptional expression levels during cerebral corticogenesis and synapse function (Ling et al., 2011).

Brain-derived neurotrophic factor (BDNF) is a growth factor that is important for supporting neuronal growth, survival, and synaptic plasticity and is implicated in learning and memory formation (Kang and Schuman, 1995; Figurov et al., 1996; Yamada et al., 2002). The lncRNA *BDNF-AS*, which is the transcribed antisense of the BDNF gene, interacts with BDNF mRNA in the brain. Silencing of *BDNF-AS* caused increased BDNF mRNA and protein levels, which resulted in neurite outgrowth and maturation, suggesting *BDNF-AS* controls the function of the BDNF gene (Lipovich et al., 2012; Modarresi et al., 2012). Moreover, *BDNF-AS* was also shown to interact with and recruit EZH2 subunit of PRC2 to the BDNF gene promoter and inhibit its expression (Pruunsild et al., 2007; Modarresi et al., 2012). All the above studies confirm that lncRNAs play important functions in synaptic plasticity and cognitive and memory processes on transcriptional and post-transcriptional levels.

## LncRNAs in neurological disorders

### LncRNAs in neurodevelopment and neurodegenerative disorders

Additionally, it acts as a critical determinant in normal brain development and neurogenesis. Recent evidence has confirmed lncRNAs as key regulatory molecules in many neurodevelopmental and neurodegenerative disorders, such as schizophrenia (Scholz et al., 2010; Li et al., 2018), autism spectrum disorder (ASD) (Wang et al., 2015), Alzheimer’s (Faghihi et al., 2010), Huntington’s (Sunwoo et al., 2017), and Parkinson’s (Ni et al., 2017) diseases. We have summarised functional lncRNAs involved in neurological disorders in Table 2.

**Table 2:** Dysregulated lncRNAs in neurological disorders.

lncRNA	Description	Associated disease	Regulation	Biological function	References
MIAT	Downregulated during neuronal activation, regulates cell proliferation, apoptosis, and migration	Schizophrenia (SCZ)	Down	Functions as a competitive endogenous RNA (ceRNA) for miRNAs and regulates signalling pathways and gene expression	Liu et al. (2018), Roberts et al. (2014), and Sun et al. (2018)
MSNP1AS	Regulates neuronal development	Autism spectrum disorder (ASD)	Up	Regulates the expression of Moesin protein that controls neurite number and length	Wilkinson and Campbell (2013)
Sox2OT	Potential biomarker of neurodegeneration	Alzheimer’s disease (AD)	Up	Regulates expression of Sox2 TF to suppress neurogenesis	Arisi et al. (2011)
BACE1-AS	Upregulated in AD brains, stabilises BACE1 mRNA, contributes to AD pathology	Alzheimer’s disease (AD)	Up	Increases BACE1 protein expression, leading to the formation of amyloid plaques through a post-transcriptional feed-forward mechanisms	Faghihi et al. (2008) and Wan et al. (2017)
HAR1	Decreased expression in HD striatum, regulates REST target genes	Huntington’s disease (HD)	Down	Transcriptional repression of REST target genes	Johnson et al. (2010)
HTTAS v1	Reduced expression in HD frontal cortex, regulates Huntingtin expression	Huntington’s disease (HD)	Down	Suppress the expression of the Huntingtin gene	Chung et al. (2011)
NEAT1	Upregulated in HD brain tissues	Huntington’s disease (HD)	Up	Implicated in the integrity of the nuclear paraspeckle assembly and gene regulation	Johnson (2012)
TUG1	Upregulated in HD brain tissues, activated by p53, interacts with PRC2	Huntington’s disease (HD)	Up	Silences downstream HD-associated genes through epigenetic regulation	Hwang and Zukin (2018)
DGCR5	Downregulated in HD brain tissues, direct target of REST	Huntington’s disease (HD)	Down	Aberrant accumulation of REST in neurons in HD	Hwang and Zukin (2018)
MEG3	1. Downregulated in HD brain tissues, direct target of REST 2. Downregulated in Glioma brain tissues	Huntington’s disease (HD) Glioma	Down	1. Aberrant accumulation of REST in neurons in HD 2. Regulates cell proliferation and promotes p53-mediated apoptosis	Hwang and Zukin (2018) and Wang et al. (2012)
lnaPINK1	Transcribed from the antisense of PINK1 locus, stabilises PINK1 expression	Parkinson’s disease (PD)	Up	Stabilise PINK1 expression resulting in disturbed mitochondrial function and increased apoptosis	Scheele et al. (2007)

#### Schizophrenia

Schizophrenia (SCZ) is a mentally debilitating disease with a wide range of neurocognitive losses. Both genetic and environmental factors are associated with the pathophysiology of SCZ (Seidman and Mirsky, 2017). Many lncRNAs have been shown to play roles in the pathogenesis of SCZ and have been discovered as biomarkers and therapeutic targets for SCZ.

For example, the expression of the lncRNA *MIAT*, also known as *Gomafu* or *RNCR2*, in SCZ was downregulated during neuronal activation (Sun et al., 2018). As mentioned above, *MIAT* can function as a competitive endogenous RNA (ceRNA) for miR-150-5p, miR-24, miR-22-3p, or miR-150 to promote cell proliferation, apoptosis, and migration. It can also participate in signalling pathways to increase Nrf2 (nuclear factor erythroid 2-related factor 2) and Oct4 expression. The following studies showed that *MIAT* can directly bind to various splicing factors, such as QKI and SRSF1, to control neuronal genes. In SCZ patient brains, *MIAT* was upregulated, which caused a suppression of SCZ-associated genes such as *DISC1* (disrupted in schizophrenia 1), *ERBB4* (V-Erb A erythroblastic leukaemia viral oncogene homolog 4), and their alternatively spliced variants (Roberts et al., 2014; Liu et al., 2018; Sun et al., 2018).

#### Autism spectrum disorder

Autism spectrum disorder (ASD) is a heterogeneous group of neurodevelopmental disorders identified by disabled social intuition, communication, and recurring stereotyped behaviours (Tang et al., 2017).

Hundreds of aberrantly expressed lncRNAs are identified by microarray expression analysis of ASD human post-mortem brain tissue (prefrontal cortex and cerebellum) compared to healthy controls. These lncRNAs are found to be transcribed in close proximity to genes known to be associated with neurodevelopmental and psychiatric diseases. It was observed that the diverse expression of lncRNAs in healthy controls was much greater than in ASD brain tissue samples (1,375 lncRNAs vs. 236 lncRNAs, respectively) (Ziats and Rennert, 2013).

Analysis of RNA-seq data identified overlapping antisense lncRNAs at 38 protein-coding loci associated with ASD. *SYNGAP1-AS* is one of these antisense transcripts that was found to be highly expressed in the ASD post-mortem prefrontal cortex and superior temporal gyrus (Velmeshev et al., 2013). In addition, the lncRNA *MSNP1AS*, encoded by the opposite strand of the moesin pseudogene 1 (MSNP1) gene, which known to control neuronal development, was identified by genome-wide association study (GWAS). *MSNP1AS* showed a significant increase in post-mortem samples, and overexpression of *MSNP1AS* negatively regulated the expression of Moesin protein and therefore resulted in a significant reduction in neurite number and length in human cultured neurons (Wilkinson and Campbell, 2013), suggesting an important role in the pathophysiology of ASD.

#### Alzheimer’s disease

Alzheimer’s disease (AD) is a neurodegenerative disease characterised by the progressive loss of neurons within the entorhinal cortex and the hippocampus (Mucke et al., 1994; Kordower et al., 2001; Knezovic et al., 2015). The pathology of AD is not clear yet. One of the known reasons is the aggregation of β-amyloid and the amyloid plaques in the brain that are produced by the BACE1 gene. Many lncRNAs have been discovered to play a role in the pathology of AD.

One of the lncRNAs that were shown to play a role is the lncRNA *Sox2OT*, Sox2 overlapping transcript, which includes within one of its introns the single-exon Sox2 gene (Fantes et al., 2003). *Sox2OT* is expressed in mouse embryonic stem cells and embryoid body differentiation (Mercer et al., 2008; Amaral et al., 2009). A study that analysed the microarray expression data of AD mouse model found that *Sox2OT* is aberrantly expressed and considered to be the best biomarker of neurodegeneration in both the early and late stages of the disease (Arisi et al., 2011).

Another lncRNA, *BACE1-AS*, a conserved non-coding antisense transcript of β-secretase 1 (*BACE1*), which is shown to be upregulated in AD brains. It increases BACE1 mRNA stability *in vitro* in human cell lines and *in vivo* in murine brains, thus upregulating BACE1 protein that causes proteolysis of APP and formation of hydrophobic β-amyloid peptide aggregates, Aβ1-42, which are the hallmarks of AD pathology (Faghihi et al., 2008). Knockdown of *BACE1-AS in vivo* resulted in the suppression of *BACE1*, *BACE1-AS*, and β-amyloid levels in the brain (Wan et al., 2017). *BACE1-AS* inhibition offers a good strategy for specifically reducing BACE1 level *in vivo*, which promises great therapeutic promise.

In addition, the lncRNA *BC200* was shown to be reduced in the frontal cortex, specifically in the neurite outgrowths of neurons of normal ageing brain, but increased in AD patients, and the severity of the disease corresponded with the increased level of *BC200* (Mus et al., 2007). Nonetheless, another group showed opposite results: BC200 RNA showed a 70% reduction in AD brains compared with the normal ones (Lukiw et al., 1992). These differences between the two studies might be caused by targeting different brain regions or timing during sampling. Yet, whether *BC200* level increased or decreased in the AD brain, its aberrant expression was detected, with the need to understand its mechanism and function in detail. More lncRNAs such as *NAT-Rad18* (Parenti et al., 2007), *17A* (Massone et al., 2011), *GDNF-AS*, and *BCYRN1* all showed to play a role in the pathophysiology of AD brains (Wan et al., 2017).

#### Huntington’s disease

Huntington’s disease (HD) is a genetic neurodegenerative disorder caused by an expansion of a CAG triplet repeat stretch within the first exon of the huntingtin gene, which results in a mutant form of the huntingtin protein (Wu et al., 2013). HD symptoms include dementia, chorea, and psychiatric instabilities and occur as an estimate in 1/10,000 people. Huntingtin has been reported to regulate the nuclear-cytoplasmic translocation of the transcriptional repressor RE1-silencing TF/neuron-restrictive silencer factor (REST/NRSF), while the mutated huntingtin gene had defective translocation of REST/NRSF, which led to the aberrant expression of REST target genes (Zuccato et al., 2003; Shimojo, 2008).

To investigate and uncover lncRNAs involved in HD, a study characterised lncRNA expression profile in human HD brain tissues in comparison to healthy controls. It revealed that the expression of the lncRNA *HAR1* was significantly decreased in the striatum. REST is a direct target of *HAR1*, which results in transcriptional repression of REST target genes (Johnson et al., 2010).

Huntingtin antisense (HTTAS) is a natural antisense transcript at the HD repeat locus. *HTTAS v1* (exons 1 and 3) are reduced in the human HD frontal cortex. Overexpression of *HTTAS v1* in cell lines reduces endogenous HTT transcript levels, while its knockdown increases HTT transcript levels. These observations confirm the existence of a gene antisense to Huntingtin that regulates its expression (Chung et al., 2011).

More lncRNAs have been shown to have abnormal expression patterns in HD brain tissues. For example, the lncRNAs *NEAT1* (nuclear paraspeckle assembly transcript 1) and *TUG1* were shown to be upregulated, while the lncRNAs *DGCR5* (DiGeorge syndrome critical region gene 5) and *MEG3* (maternally expressed 3) were shown to be downregulated. In the pathophysiology of HD, the lncRNA *TUG1* is activated by p53 and then interacts with the epigenetic silencer polycomb repressive complex 2 (PRC2), therefore silencing downstream HD-associated genes. While the lncRNAs *DGCR5* and *MEG3* are both direct targets of REST, when they are downregulated, REST becomes aberrantly accumulated in the neurons in HD (Johnson, 2012; Hwang and Zukin, 2018).

#### Parkinson’s disease

Parkinson’s disease (PD) is a chronic neurodegenerative disease caused by defects in dopamine-producing cells that lead to a loss of motor abilities.

Scientists have been studying the disease for years, yet the pathophysiology of the disease has not been understood yet. PD-related genes have been discovered, for example, α-synuclein, Parkin, PINK1 (phosphatase and tensin homologue-induced putative kinase 1), LRRK2 (leucine-rich repeat kinase 2), and DJ-1 (also known as Parkinson disease protein 7 [PARK7]). These genes are known to be associated with mitochondrial function, suggesting the homeostasis properties of mitochondria play an important role in the disease (Sai et al., 2012).

PINK1 gene is controlled by the tumour suppressor PTEN (phosphatase and tensin homolog). Aberrant PINK1 expression causes defective mitochondrial function, dopamine release, and motor deficits (Morais et al., 2009). The *lnaPINK1* is transcribed from the antisense of PINK1 locus, and it functions to stabilise PINK1 expression. Knockdown of *lnaPINK1* causes inhibition of PINK1 in neurons, suggesting that both of them are concordantly regulated during mitochondrial biogenesis and proposing a strategy for treating PD through regulation of the PINK1 locus (Scheele et al., 2007). More lncRNAs, such as *Huc1* and *Huc2*, *H19* upstream conserved 1 and 2, *lincRNA-p21*, *MALAT1*, *SNHG1*, and *TncRNA*, are all abnormally expressed in the PD brain (Kraus et al., 2017). Studies showed that they are associated with proliferation, synaptogenesis, and apoptosis. Importantly, their aberrant expression precedes PD, which suggests that they could be used as biomarkers of PD (Kraus et al., 2017).

#### Traumatic brain injury and cerebral haemorrhage

LncRNAs have emerged as critical regulators in the pathophysiology of traumatic brain injury (TBI) and cerebral haemorrhage. These non-coding transcripts participate in diverse cellular processes and molecular pathways, influencing neuroinflammation, cell death, angiogenesis, and tissue repair. In TBI, lncRNAs such as *MALAT1*, *NEAT1*, and *H19* have been implicated in modulating neuronal apoptosis, glial activation, and blood–brain barrier integrity (Xin and Jiang, 2017; Zhong et al., 2017; Chen Z. et al., 2019). In cerebral haemorrhage, lncRNAs like *MIAT* and *PVT1* have been associated with vascular damage, haematoma resolution, and neuronal survival (Li E. Y. et al., 2019; Gong and Wei, 2024).

All the above studies investigated in different diseases confirm that lncRNAs are playing major roles in the pathophysiology of neurodevelopmental and neuropsychiatric diseases, yet with poorly understood aetiologies. The use of these studies to develop effective diagnostic and therapeutic methods should be considered cautiously since it has not been fully investigated until now how any aberrant expression of these lncRNAs can be mechanistically involved in the disease pathology rather than just being a marker of the disease. Moreover, data from large *in vivo* human sample cohorts controlling for disease severity and comorbidities are understated in these studies.

### LncRNAs in glioma development

A glioma is a type of tumour that arises from glial cells in the brain. It is identified by uncontrolled cell growth, necrosis, and dynamic angiogenesis, with symptoms including headache, seizures, impaired neurological function, and, eventually, death (Ferris et al., 2017; Srinivas et al., 2023). Current non-invasive techniques (computed tomography [CT], magnetic resonance imaging [MRI], or positron emission tomography [PET] scans) can help in identification and localisation of these tumours, yet we cannot characterise their pathology by these techniques alone. High-grade gliomas can infiltrate into the extracellular matrix of the brain, which also makes it hard to do surgery and radiotherapy on them (Gwak et al., 2012; Park J. Y. et al., 2014). Hence, identifying the molecular mechanisms and key regulators underlying gliomagenesis is important for the cure of this disease.

Multiple studies have identified some differently expressed lncRNAs that contribute to the pathogenesis of glioblastoma multiform (Ellis et al., 2012). For example, the lncRNA *MEG3*, a maternally expressed gene 3, has been shown to be downregulated in glioma brain tissues compared to normal ones (Wang et al., 2012). Overexpression of the lncRNA *MEG3 in vitro* in human glioma cell lines negatively affects cell proliferation and promotes p53-mediated apoptosis. Another example is the lincRNA *H19* and its derivative miR-675, which was shown to play a role in glioma cell invasion (Shi et al., 2014). Furthermore, lncRNAs such as *FOXD2-AS1*, forkhead box D2 adjacent opposite strand RNA 1, *HOTTIP*, homeobox A (HOXA) distal transcript antisense RNA, and *HOTAIR*, HOX anti-sense intergenic RNA, have also been identified as regulators of glioma progression as they play roles in cell cycle and epigenetic modifications (Pandey et al., 2014; Vance et al., 2014; Latowska et al., 2020; Chen et al., 2021). All these lncRNAs were confirmed to regulate glioma development and can be considered as potential drug targets in glioma treatment.

## Biomarkers and therapeutic targets

Given the proven functional roles of lncRNAs in the brain, the idea of using them for diagnostic and therapeutic benefit arises. Below, we discuss different methods that suggest the use of lncRNAs in neurological disorder diagnosis and treatment.

### LncRNAs as biomarkers in neurological disorders

lncrnas are associated with various neurological disorders, tumours, and psychiatric conditions, which suggest that they could be used for diagnostic purposes as biomarkers precise to a specific disease and can be sensitive and accurate to early and rapid detection.

Many of the lncRNAs mentioned in this review can be used as biomarkers using minimally invasive methods. For example, *in vivo* analysis of plasma-derived circulating RNAs has confirmed the lncRNA *BACE1-AS* as a diagnostic marker of AD (Fotuhi et al., 2019; Srinivas et al., 2023). Moreover, identifying lncRNA as biomarkers with high specificity and sensitivity from the bloodstream at sites distal to the brain has also been done in other neurological disorders, and some of them were confirmed by imaging and tissue biopsies (Srinivas et al., 2023). However, although identifying lncRNA expression in the bloodstream by isolation of serum, plasma, leukocytes, or exosomes is helpful and informative, it may not give a full representation of the molecular changes accruing in the brain. Therefore, samples from the CSF, which are in contact with the brain, have been used to identify circulating lncRNA expression, and it has been considered as a more specific biomarker for diagnosing certain brain pathologies (Hossein-Nezhad et al., 2016; Pan et al., 2020; Whitlock et al., 2022). For example, the lncRNA *MALAT1* was found to be elevated in the CSF of patients with AD compared to healthy controls, suggesting its potential as a diagnostic marker (Zhuang et al., 2020). Additionally, the long non-coding RNA activated by TGF-β (lncRNA-ATB) expression was shown to be significantly upregulated in the CSF of AD (Wang et al., 2018).

However, it is important to emphasise that even though the above-mentioned methods are helpful and promising, relying on circulating lncRNAs as biomarkers for brain disorders is difficult. qRT-PCR is used to identify the expression of lncRNAs in the bloodstream, but there is no known reference for lncRNAs from different sources (e.g., plasma vs. serum vs. CSF). In addition, most lncRNAs are expressed at low levels, making it difficult to detect them. Importantly, lncRNAs might not be specifically dysregulated in one disease and could be expressed in more than one. Like the lncRNA *NEAT1*, which was found to be aberrantly expressed in PD, AD, and ALS (An et al., 2018). Because of that, liquid biopsies can be used in addition to existing diagnostic methods rather than as a defective diagnostic alone. Interestingly, the expression of some lncRNAs that cross the blood–brain barrier (BBB) and become enriched in cerebrospinal fluid (CSF) is high in gliomas *ex vivo*, making them possible biomarkers for brain tumours (Xu et al., 2021).

### LncRNAs as therapeutic targets for neurological disorders

**Figure 4:**
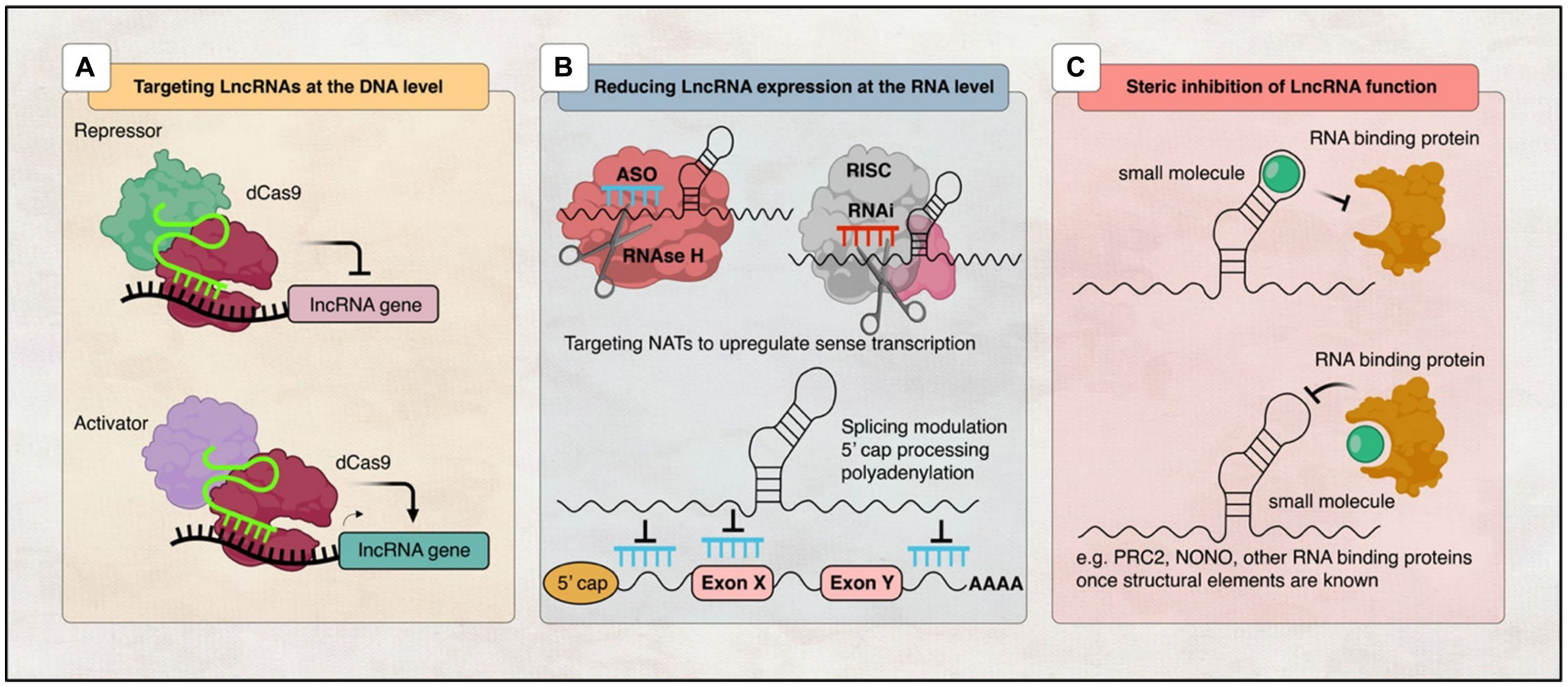
Illustration of various strategies employed to target lncRNAs. **(A)** DNA editing: CRISPRi and CRISPRa tools enable transcriptional silencing or activation of lncRNA-expressing loci, respectively. **(B)** Modulation of RNA levels: ASOs and siRNAs can be utilised to decrease lncRNA levels, thereby modifying the expression of associated protein-coding genes. Additionally, recruitment of RBPs can mediate RNA processing events such as 5′ capping, splicing, or polyadenylation to regulate lncRNA expression, while RNAi induces RISC-mediated cleavage of the lncRNA transcript. **(C)** Steric inhibition: Small molecules target secondary and tertiary structures of lncRNAs and/or their binding partners, impeding their interaction.

In addition to their use as biomarkers, lncRNAs have also been studied as potential *in vivo* therapeutic targets for the treatment of neurological disorders (Wahlestedt, 2013; Roovers et al., 2018). Many lncRNA-based therapies have been discovered to target lncRNA transcripts for degradation or interreference, and some pharmaceutical companies are also keenly developing lncRNA-targeting therapeutics (Li and Chen, 2013; Ling et al., 2013; Park J. Y. et al., 2014). So far, there are 11 RNA-based therapies that have been approved by the U.S. Food and Drug Administration (FDA) and/or the European Medicines Agency (EMA). The only one that targets the brain is Nusinersen, which is used to treat spinal muscular atrophy, while the others target other tissue types (Winkle et al., 2021; Figure 4).

#### Targeting lncRNAs at the DNA level

Newly discovered genomic editing methods like CRISPR technology have provided the possibility of understanding the lncRNA mechanism of action. It allowed us to interfere with gene expression and therefore to silence or activate lncRNA transcription (Jinek et al., 2012; Cong et al., 2013; Mali et al., 2013; Policarpo et al., 2021; Qi et al., 2021). One way to achieve this is to use the Cas9 approach, in which a mutant form of Cas9 without endonuclease activity binds to transcription repressors or activators to silence or activate the transcription of specific genes (Liu S. J. et al., 2017; Arun et al., 2018; Figure 4A). This technique was shown to be successful in a mouse model in improving the phenotype of Angelman Syndrome, a rare genetic condition that affects the nervous system and causes severe physical and learning disabilities. It reduced the expression of *UBE3A-ATS* and activated the paternal *UBE3A* using adeno-associated viral (AAV) delivery system, which provided evidence that this method is therapeutically significant (Wolter et al., 2020). Moreover, another AAV-based therapy targeting *SMN* gene, called Onasemnogene abeparvovec, is the first gene therapy for SMA approved in the United States and proves that AAV methods are effective in treating neurological diseases (Hoy, 2019). Even though recent developments in CRISPR methods have great possibilities as therapeutic targets for neurological diseases, there are still many challenges along the way that need to be resolved before their use. For example, finding an efficient delivery method for the CNS and the ability to reverse DNA editing pose risks due to DNA on-target and off-target effects (Sun and Roy, 2021). In addition, most lncRAs, upon CRISPR-mediated targeting, are at risk of accidentally effecting the expression of neighbouring genes (Goyal et al., 2017). Therefore, it is important to continue investigating and improving these therapeutic techniques for better treatment.

#### Targeting lncRNAs at the RNA level

The ability to interfere with RNA expression using oligonucleotides has been proven to be a good strategy to affect any target RNA transcript (Arun et al., 2018). Currently, there are two major methods using oligonucleotide-based therapies: antisense oligonucleotides (ASOs) and RNA-mediated interference (RNAi), which have the same principle of using their catalytic activity to bind with their target RNA *via* Watson-Crick base pairing (Watts and Corey, 2012).

ASOs are single-stranded nucleotide sequences that bind target RNA transcripts to either affect splicing events such as 5′-cap formation, splicing, and polyadenylation, trigger RNase H cleavage, or inhibit translation (DeVos and Miller, 2013). Alternatively, siRNAs are short complementary hybrid RNA strand sequences that use the cellular microRNA machinery to inhibit the translation of the target RNA (Hannon and Rossi, 2004; Figure 4B). However, it is important to mention that lncRNAs are predominantly localised in the nucleus, and thus siRNAs may not be accessible to lncRNAs like mRNAs in the cytoplasm. Yet, in many studies, researchers were able to knock down lncRNAs regardless of their subcellular localisation (Park J. Y. et al., 2014). So far, antisense oligonucleotides have advantages over siRNAs, including their high specificity and low off-target effects. Recent studies confirmed a successful depletion of the lncRNA *MALAT1* in mouse lung cancer cells by using ASOs (Wilusz et al., 2008; Tripathi et al., 2010, 2013).

Until now, no RNA-based therapies for targeting lncRNA in humans have been approved. Only ASO therapy showed some promising results *in vivo* for the degradation of natural antisense lncRNA transcripts (NATs) in the brain. ASOs that inhibit NAT expression (AntagoNATs) have been shown to reduce transcription of the gene encoding BDNF while enhancing neuronal outgrowth (Modarresi et al., 2012). ASOs also increased the expression of wild-type sodium voltage-gated channel alpha subunit 1 (SCN1A) gene, which is known to be mutated in Dravet syndrome, a rare genetic brain disease characterised by lifelong epilepsy (Hsiao et al., 2016). Moreover, the use of AntagoNATto suppressed the gene *UBE3A-AS*, which is known to suppress the paternal copy of the ubiquitin protein ligase E3A gene (UBE3A), which improves cognitive deficits of the Angelman Syndrome in mice model (Meng et al., 2015).

In addition, some lncRNAs play a protective or restorative role in diseases, and upregulating their expression is useful for treatment. For example, the lncRNA *GDNF*, glial cell-derived neurotrophic factor, is known to enhance the survival of dopaminergic neurons and could therefore improve the symptoms of PD. Another example is *SINEUPs*, a class of antisense lncRNAs that promote mRNA translations and can be used to produce proteins. Overexpression of GDNF-targeting *SINEUPs* in the mouse striatum increases the level of GDNF protein and dopamine while reducing motor defects and neurodegeneration (Espinoza et al., 2020).

Importantly, even though scientists have been recently considering the use of lncRNAs as therapeutic targets in various tissues and diseases, due to their complexity, the brain has acquired certain challenges. First, it is difficult for the administered therapy to cross and penetrate the blood–brain barrier (BBB) without the use of risky intrathecal or intracerebroventricular injection methods. Researchers have lately developed MIND, a minimally invasive nasal depot method that can deliver antagonists against the lncRNA *BDNF-AS* through the olfactory bulb into the mouse brain (Padmakumar et al., 2021). Therefore, it is important that the developed therapies cross the cell membrane, be cell subtype- and sequence-specific, and have low off-target effects, low toxicity, and immunogenicity. Another way to achieve this is through the use of exosomes or other vesicles as therapeutic vehicles. One group was able to deliver glycoprotein-circSCMH1 through injection of an extracellular vesicle, which has improved the neuronal plasticity and recovery of mice with cerebral occlusion without toxicity or immunogenic response (Yang et al., 2020). These new methods may bring in the near future important developments in disease-specific lncRNA therapeutic targeting.

#### Inhibition of lncRNAs function

Another therapeutic approach that has been proposed is to interfere with the functions of lncRNAs instead of effecting their expression on DNA/RNA levels. Many studies confirmed that lncRNAs function through interaction with RBPs and protein complexes, which suggest the use of ASOs or small molecules to disrupt these interactions (Meyer et al., 2020; Figure 4C).

The recent development of new sequencing and structure assays such as SHAPE (Wilkinson et al., 2006), SHAPE-MaP (Smola et al., 2015), PARIS (Lu et al., 2016), or CROSSalign (Delli Ponti et al., 2018) allowed scientists to map the secondary and tertiary structural domains of lncRNAs that interact with proteins (Smith et al., 2013; Mondal et al., 2015; Somarowthu et al., 2015; Liu F. et al., 2017; McCown et al., 2019; Balas et al., 2021). For example, the lncRNA *MALAT1* has a triple helix at its 3′ end, and targeting this structure reduced *MALAT1* levels in cells, which suggest an important role for this structure in controlling the expression of *MALAT1* (Brown et al., 2014, 2016). Another example is the lncRNA *AS-Uchl1*, in which inhibiting its short hairpin motif eliminated *AS-Uchl1*’s ability to upregulate UCHL1 protein levels (Podbevsek et al., 2018). In addition, the RBP NONO was shown to be specifically binding to conserved motifs in the lncRNA *NEAT1* (Simko et al., 2020). These motifs are known and recognised by PRC2 (Wang et al., 2017), which is known to be an important binding partner for many lncRNAs, including *HOTAIR* (Rinn et al., 2007) and *XIST* (Bousard et al., 2019).

In conclusion, although the field of targeting lncRNAs using small molecules is still in the beginning and needs to be further developed, it is promising to help treat many neurological diseases (Pedram Fatemi et al., 2015; Abulwerdi et al., 2019; Donlic et al., 2019; Ren et al., 2019; Simko et al., 2020). We believe that further understanding the mechanism of actions and functional roles of lncRNAs will pave the way to transform lncRNAs, originally considered “junk” DNA, into therapeutic targets for patients affected by neurological disorders. In the meantime, several small molecules that can target another class of RNA other than lncRNAs, like miRNAs, have been developed and approved by the FDA. For example, Risdiplam was FDA approved in 2020 for the treatment of SMA, and Branaplam is undergoing clinical trial as a therapy for SMA and HD (ClinicalTrials.gov/, ID: NCT02268552, 2023). They both function to increase SMN protein levels by acting as SMN2 splicing modulators (Meyer et al., 2020). Another small group of molecules is under investigation as therapeutic agents for neurological disorders, for example, α-synuclein for PD (Zhang et al., 2020).

## Conclusion

Research in the field of lncRNAs has revealed their important function in brain development and disease. These non-coding transcripts, once considered as noise, have appeared as key players in controlling gene expression and affecting many cellular and molecular mechanisms in the brain. Dysregulation of lncRNAs has been shown to be involved in many neurological disorders, including neurodevelopmental disorders, neurogenerative diseases, and cancer. Through their interaction with chromatin modifiers, TFs, and other regulatory molecules, lncRNAs employ fine-tuning regulation over neurogenesis, neuronal differentiation, synaptogenesis, and other important brain development events. Moreover, their aberrant expression has been associated with disrupted neuronal connectivity, impaired synaptic plasticity, and abnormal gene expression patterns observed in many neurological diseases. All of this highlights the potential application of lncRNAs as diagnostic biomarkers and therapeutic targets for such disorders.

However, several challenges remain in the field of lncRNA. One challenge is identifying the downstream targets of lncRNAs, as they can interact with many molecules in a wide range of pathways. This complexity makes it difficult to use lncRNAs as specific diagnostic biomarkers and therapeutic targets for neurological disorders. Another challenge relies on investigating and unravelling the complicated mechanisms by which lncRNAs function in the brain. Researchers need to develop advanced genetic tools and animal models to understand the regulatory network of interactions of these lncRNAs with other epigenetic modifications. Moreover, extensive validation of lncRNA functions *in vivo* needs to be carried out to provide insights into their roles in brain development and disease, yet it remains difficult to identify specific targets and claim causality.

Despite these challenges, future research on lncRNAs in neurological diseases holds great promise. It will prioritise the identification and validation of lncRNA as diagnostic biomarkers, the functional characterisation of disease-associated lncRNAs, understanding lncRNA interactions, exploring therapeutic targeting of lncRNAs, and conducting functional studies in human brain tissue. Collective efforts among researchers, clinicians, and industry stakeholders are important to overcome any challenges and translate the knowledge gained from lncRNA research into effective diagnostic tools and innovative treatments for neurological disorders.

## Author contributions

FA: Writing – original draft, Writing – review & editing. EA-H: Writing – review & editing. AA: Writing – review & editing. NA: Writing – review & editing. BA-S: Writing – review & editing.
